# Coding Complete Genome Sequence of the SARS-CoV-2 Virus Strain, Variant B.1.1, Sampled from Kazakhstan

**DOI:** 10.1128/mra.01114-22

**Published:** 2022-11-14

**Authors:** Yerbol Burashev, Bekbolat Usserbayev, Lespek Kutumbetov, Yergali Abduraimov, Markhabat Kassenov, Aslan Kerimbayev, Balzhan Myrzakhmetova, Aibarys Melisbek, Meirzhan Shirinbekov, Saken Khaidarov, Edan R. Tulman

**Affiliations:** a Research Institute for Biological Safety Problems (RIBSP), Gvardeyskiy, Kazakhstan; b Faculty of Biology and Biotechnology, Al-Farabi Kazakh National University, Almaty, Kazakhstan; c Department of Pathobiology and Veterinary Science and Center of Excellence for Vaccine Research, University of Connecticut, Storrs, Connecticut, USA; Queens College CUNY

## Abstract

This article describes the results of sequencing and analysis of the entire genome of the SARS-CoV-2 virus sampled in Kazakhstan in 2021. The whole-genome sequence of the strain was 29,751 bp. According to the results of phylogenetic analysis (according to the Pangolin COVID-19 database), the SARS-CoV-2/human/KAZ/B1.1/2021 strain studied here was assigned to variant B.1.1.

## ANNOUNCEMENT

COVID-19 is an acute respiratory infection caused by the SARS-CoV-2 virus, belonging to the *Coronaviridae* family and the *Betacoronavirus* genus. Variants of the SARS-CoV-2 coronavirus have continually emerged due to the ongoing transmission and evolution of this virus around the world. Since the pandemic was first declared by the World Health Organization (WHO) in March 2020 (1), there have been outbreaks associated with variants of concern (VOC) as classified by the WHO, including B.1.17 (Alpha), B.1.351 (Beta), P.1 (Gamma), B.1.617.2 (Delta), and B.1.1.529 (Omicron) (2–5).

In Kazakhstan, the first case of human infection with coronavirus infection COVID-19 was registered in March 2020 (6). According to the Johns Hopkins University database, as of 21 October 2022, 1,484,400 confirmed cases of the disease were registered in the Republic of Kazakhstan, of which 19,052 had fatal outcomes (7).

The SARS-CoV-2/human/KAZ/B1.1/2021 *s*train was obtained from the Scientific and Practical Center for Sanitary and Epidemiological Expertise and Monitoring branch of the Republican state enterprise on the right of economic use, National Center for Public Health, Ministry of Health, Republic of Kazakhstan. Nucleic acids were extracted from the test sample using a QIAamp viral RNA minikit (Qiagen, Germany) according to the manufacturer’s protocol. Reverse transcription was performed using the SuperScript VILO cDNA synthesis kit (Invitrogen, USA). For amplification to cover the entire genome of the virus, 65 primer pairs were designed using the online Primer-BLAST program (http://www.ncbi.nlm.nih.gov/tools/primer-blast) in order to generate amplicons ranging in size from 600 to 750 bp and tiled to overlap by about 100 bp. These amplicons were generated by PCR and visualized by 1.2% agarose gel electrophoresis (Sigma, USA). PCR amplicons were purified using the PureLink PCR purification kit (Thermo Fisher Scientific, USA). Purified amplicons were sequenced using the Sanger dideoxy method using an AB3130xl (Hitachi Applied Biosystems) 16-capillary genetic analyzer autosequencer with the BigDye Terminator 3.1 cycle sequencing kit (ABI, Foster City, CA, USA). Raw chromatograms were collected using Sequencher version 5 (Gene Codes Corp.).

The MEGA X program was used for genome assembly according to default parameters (8).

The assembled complete genome of the SARS-CoV-2/human/KAZ/Britain/2021 strain is 29,751 nucleotides long. The GC content was 37.95%, and the query coverage amounted to 99%. The genome sequence was analyzed by the Pangolin COVID-19 database (https://pangolin.cog-uk.io) and belonged to the B.1.1 lineage. The sequences were published on 20 October 2022 in the NCBI GenBank database (accession no. OP684305). The 53 nucleotide mutations relative to the Wuhan reference sequence are presented in Table 1.

**TABLE 1:** Nucleotide mutations of the SARS-CoV-2/human/KAZ/B1.1/2021 SARS-CoV-2 virus strain, linage B.1.1, compared with the Wuhan-Hu-1 SARS-CoV-2 reference sequence (GenBank accession no. NC_045512)

Mutation no.	Nucleotide position	Gene	Reference base (GenBank accession no. MN908947)	Nucleotide change
1	106	5′ UTR	C	T
2	241	5′ UTR	C	T
3	344	ORF1ab	C	T
4	2530	ORF1ab	A	G
5	3037	ORF1ab	C	T
6	4449	ORF1ab	C	A
7	4455	ORF1ab	C	T
8	4475	ORF1ab	C	T
9	5829	ORF1ab	A	C
10	9749	ORF1ab	A	G
11	9867	ORF1ab	T	G
12	10198	ORF1ab	C	T
13	11289	ORF1ab	C	
14	11290	ORF1ab	T	
15	11291	ORF1ab	G	
16	11292	ORF1ab	G	
17	11293	ORF1ab	T	
18	11294	ORF1ab	T	
19	11295	ORF1ab	T	
20	11296	ORF1ab	T	
21	11297	ORF1ab	A	
22	14408	ORF1ab	C	T
23	15017	ORF1ab	C	T
24	20759	ORF1ab	C	T
25	21080	ORF1ab	A	G
26	21446	ORF1ab	A	G
27	21646	S	C	T
28	21648	S	C	T
29	21784	S	T	A
30	21789	S	C	T
31	21846	S	C	T
32	23014	S	A	C
33	23403	S	A	G
34	23520	S	C	T
35	23751	S	C	T
36	23997	S	C	T
37	24000	S	G	T
38	24538	S	A	T
39	25688	ORF3a	C	T
40	26110	ORF3a	C	T
41	27015	M	G	T
42	27389	ORF6	C	T
43	27630	ORF7a	C	T
44	27667	ORF7a	G	A
45	27739	ORF7a	C	T
46	28881	N	G	C
47	28882	N	G	A
48	28883	N	G	A
49	29436	N	A	T
50		3′ UTR		T
51		3′ UTR		A
52		3′ UTR		C
53		3′ UTR		T

**FIG 1:**
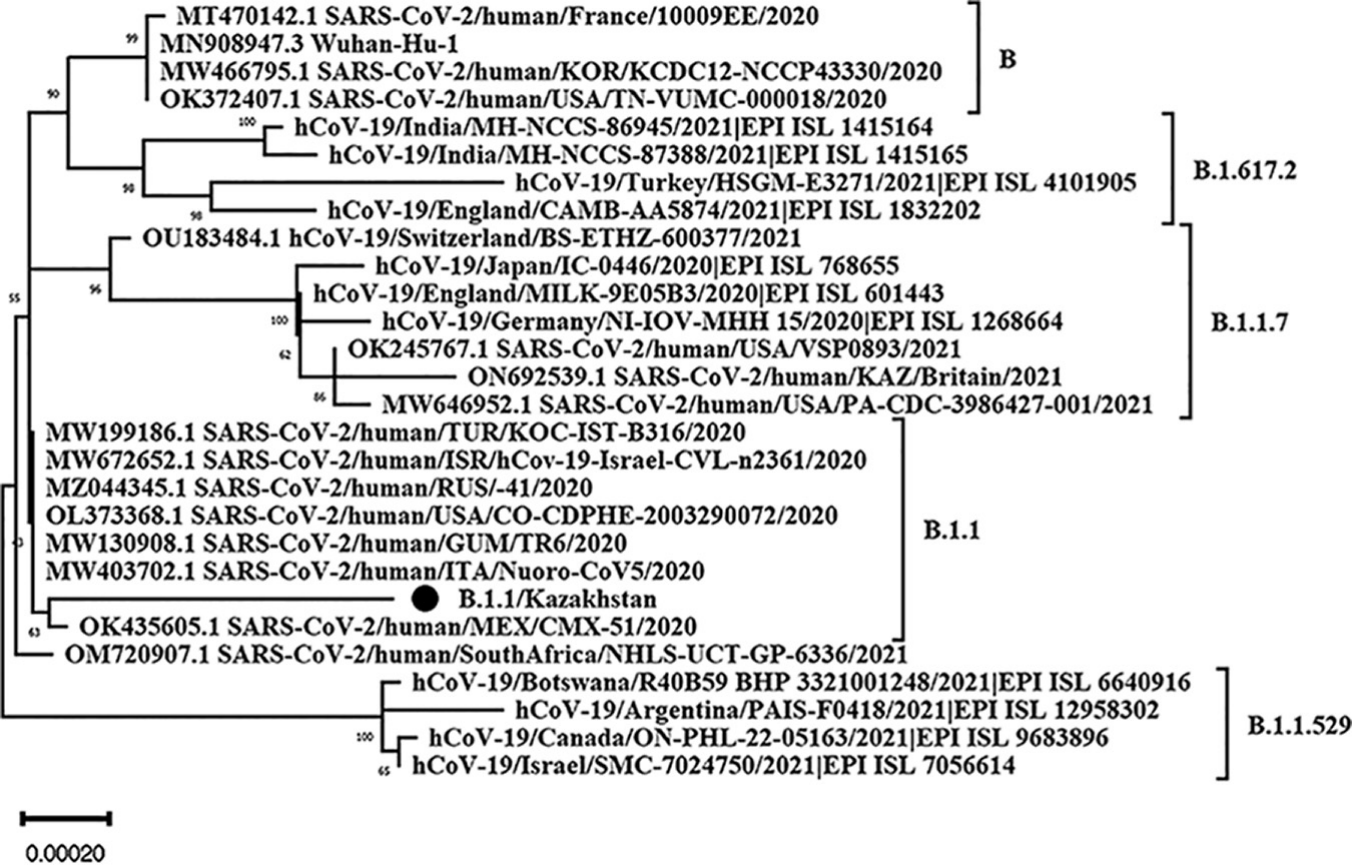
Phylogenetic analysis of the SARS-CoV-2/human/KAZ/B1.1/2021 isolate. The phylogeny was generated using the neighbor-joining method (9). The optimal tree is shown. The percentage of replicate trees in which the associated taxa clustered together in the bootstrap test (500 replicates) are shown next to the branches (10). The tree is drawn to scale, with branch lengths the same units as those of the evolutionary distances used to infer the phylogenetic tree. The evolutionary distances were computed using the Kimura 2-parameter method (11) and represent the estimated number of base substitutions per site. This analysis involved 28 nucleotide sequences. Codon positions included are 1st, 2nd, 3rd, and noncoding. All positions containing gaps and missing data were eliminated (complete deletion option). There were a total of 25,145 positions in the final data set. Evolutionary analyses were conducted in MEGA 11 (12).

According to the results of phylogenetic analysis, the strain SARS-CoV-2/human/KAZ/B1.1/2021 (Fig. 1, black circle) analyzed here clustered with the strains of the clade B.1.1 isolated in Europe, the Middle East, and America isolated in 2020; however, the genetic distance indicates divergence of SARS-CoV-2/human/KAZ/B1.1/2021 relative to B.1.1 strains isolated in 2020.

Phylogenetic analysis was performed using MEGA 11. In Fig. 1, the *x* axis represents the scale of the tree. A scale bar with a value of 0.00020 is shown at the bottom.

### Data availability.

The complete nucleotide sequence of the SARS-CoV-2/human/KAZ/Britain/2021 strain was deposited to GenBank under accession no. OP684305.
